# Supplementary material for: Global site-specific N-glycosylation analysis of HIV envelope glycoprotein
Source: Nat Commun. 2017 Mar 28;8:14954. doi: 10.1038/ncomms14954 (PMC5379070; doi:10.1038/ncomms14954)
Supplement: Supplementary Information — Supplementary Figures and Supplementary Tables. [file ncomms14954-s1.pdf]

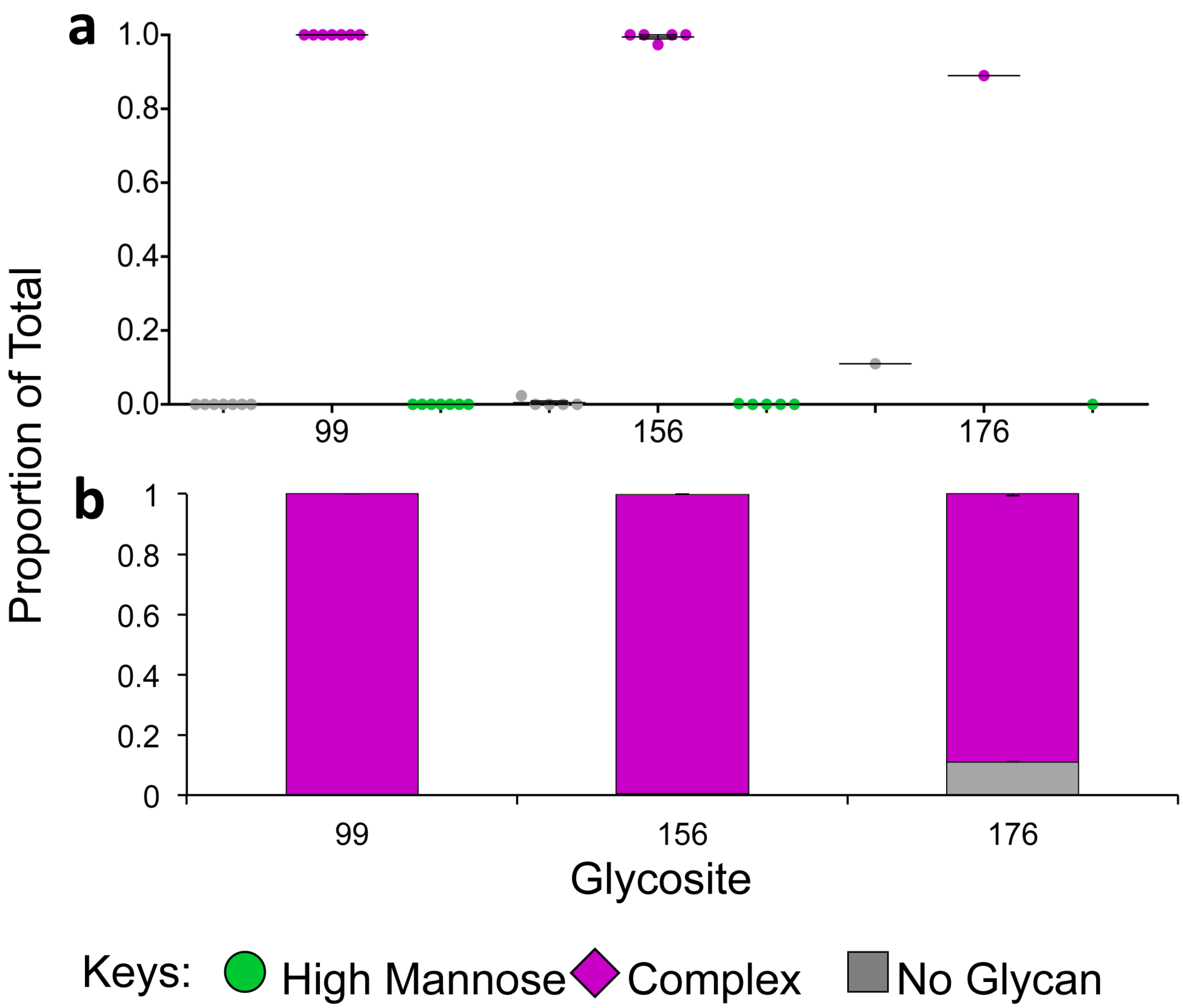

Supplementary Figure 1

Site-specific glycosylation of bovine fetuin. (a) Scatter plot of the site-specific glycosylation of bovine fetuin. A set of peptides with N+0 (grey dots), N+3 (purple dots), and N+203 (green dots) modifications were displayed only when at least one of the three had a peak area of at least > 5E8. (b) Color-coded bar graph of the site-specific glycosylation of bovine fetuin. Mean ± SEM were plotted.

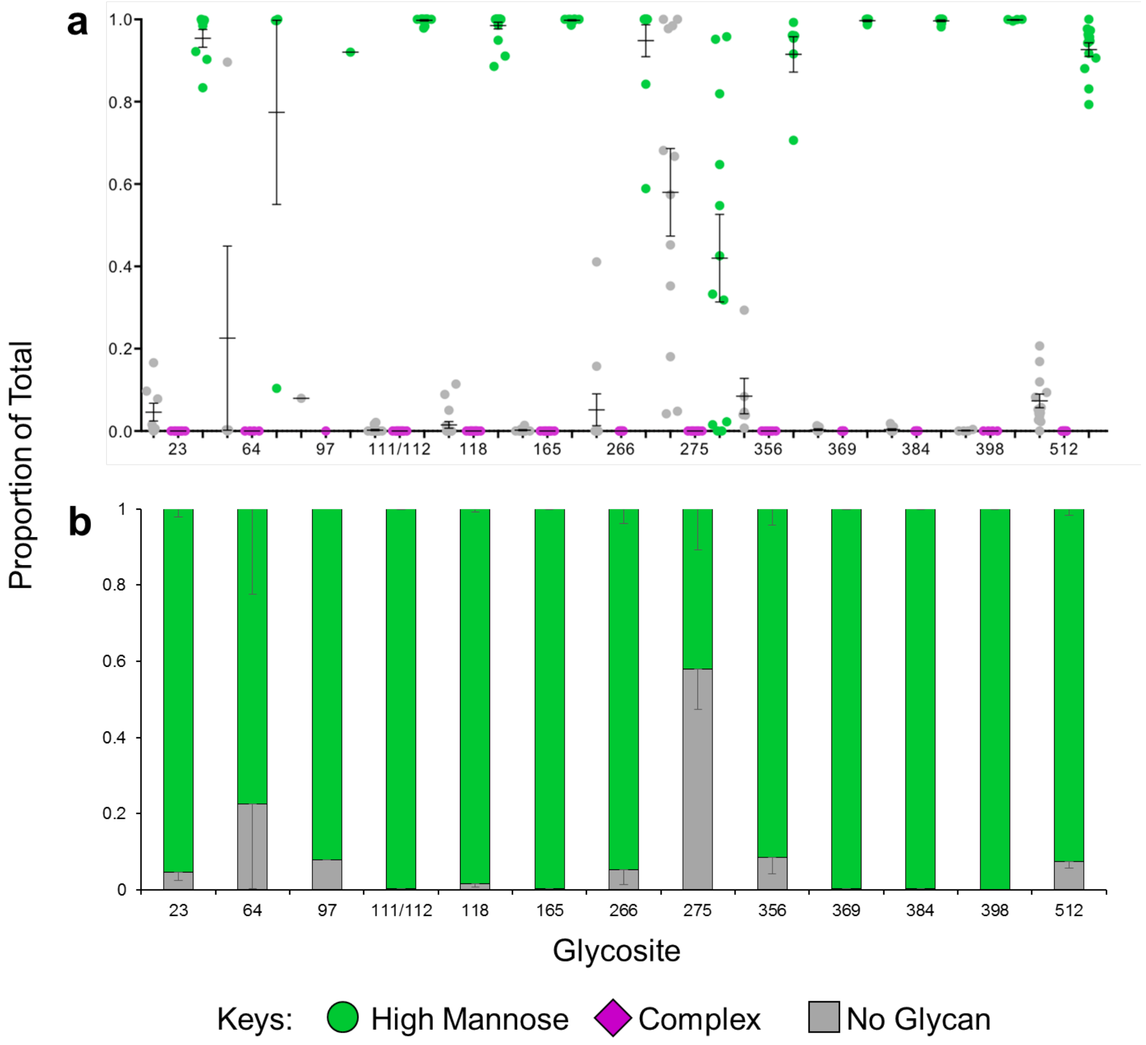

Supplementary Figure 2

Site-specific glycosylation of invertase produced by the yeast *S. cerevisiae*. (a) Scatter plot of the site-specific glycosylation of invertase. A set of peptides with N+0 (grey dots), N+3 (purple dots), and N+203 (green dots) modifications were displayed only when at least one of the three had a peak area of at least > 5E8. (b) Color-coded bar graph of the site-specific glycosylation of invertase. Mean ± SEM were plotted.

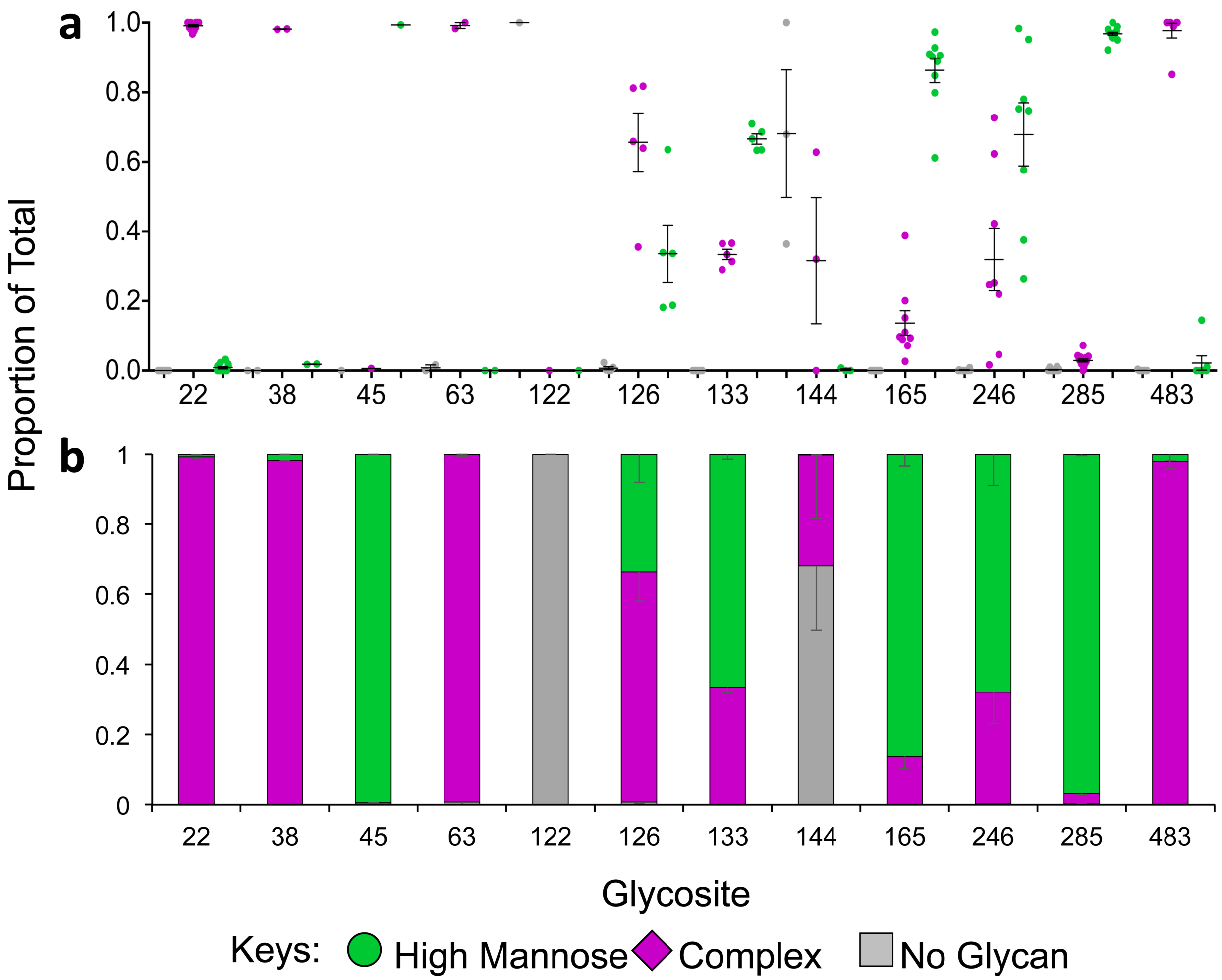

Supplementary Figure 3

Site-specific glycosylation of the recombinant influenza virus hemagglutinin from H3N2 strain A/Victoria/361/2011 produced in HEK 293 F cells. (a) Scatter plot of the site-specific glycosylation of the hemagglutinin. A set of peptides with N+0 (grey dots), N+3 (purple dots), and N+203 (green dots) modifications were displayed only when at least one of the three had a peak area of at least > 5E8. (d) Color-coded bar graph of the site-specific glycosylation of the hemagglutinin. Mean ± SEM were plotted.

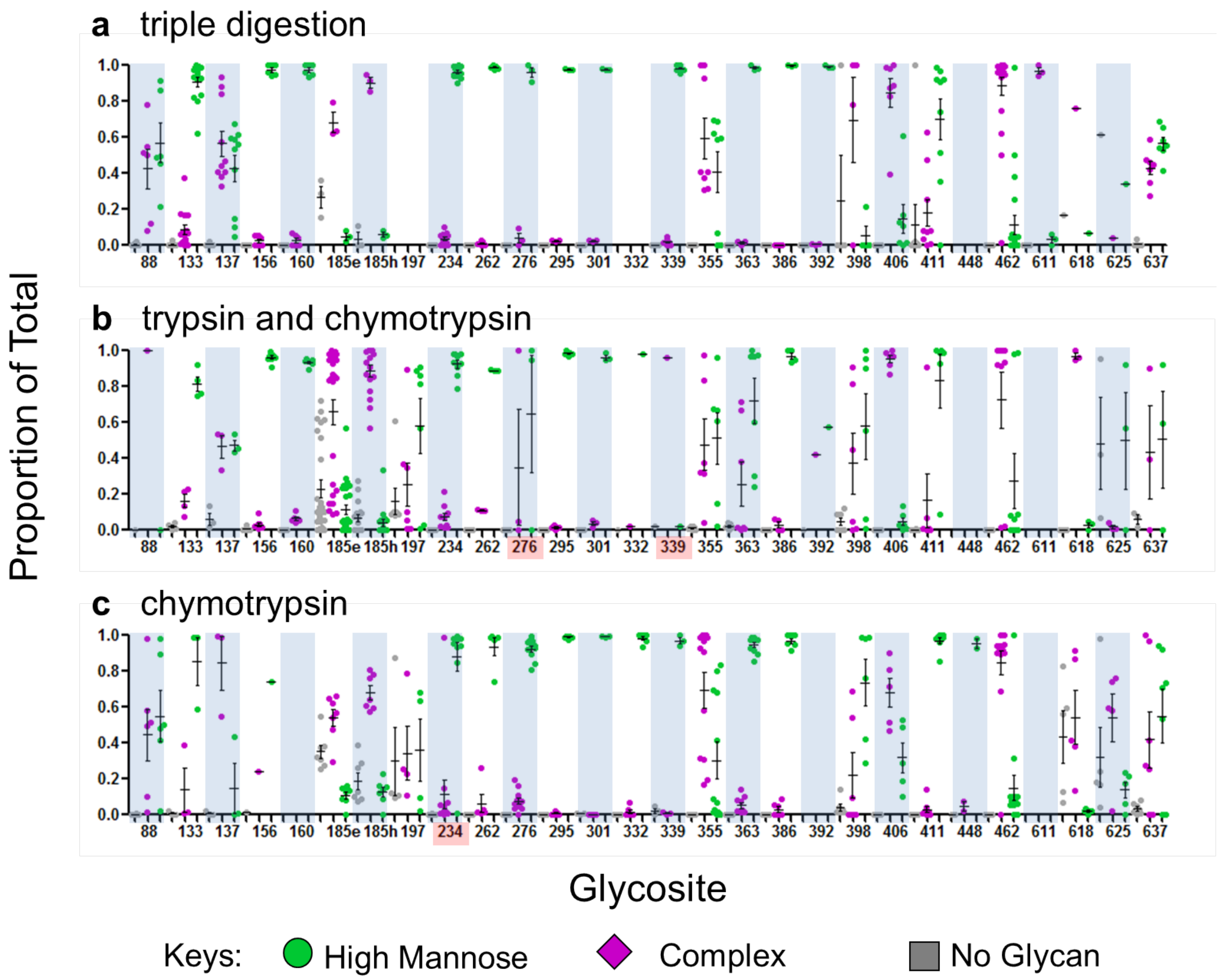

Supplementary Figure 4

Scatter plot of the site-specific glycosylation of BG505 SOSIP.664 trimer with a breakdown on the used proteases: (a) triple digestion, (b) the combination of trypsin and chymotrypsin, (c) chymotrypsin, related to Fig. 1c. Those glycosites that showed biases were highlighted in light coral.

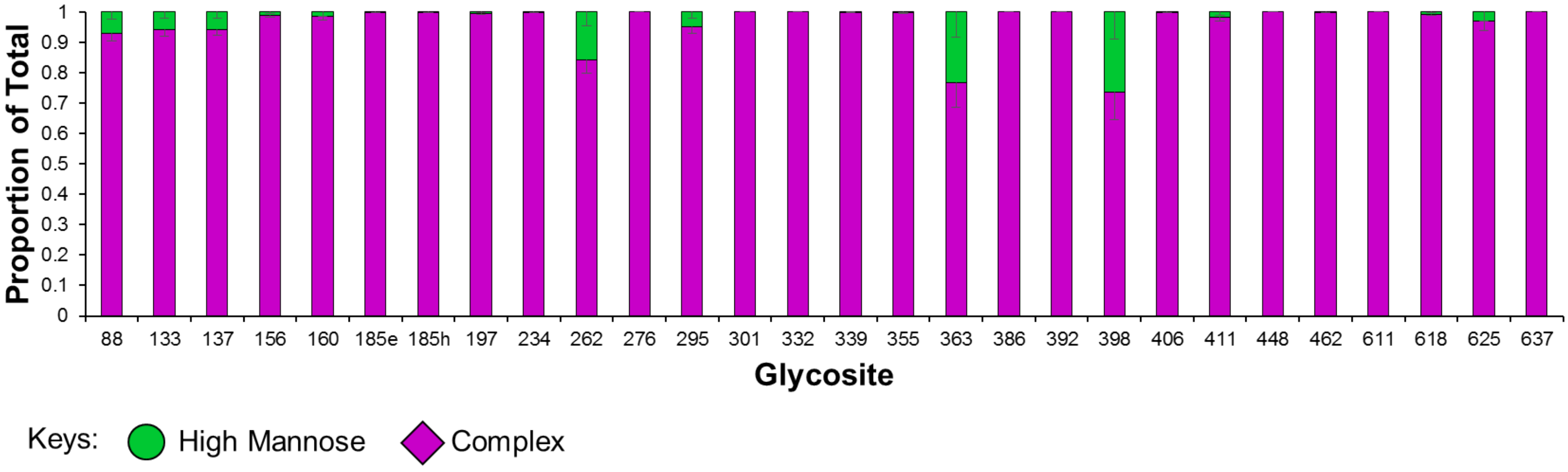

Supplementary Figure 5

Validation of PNGase F treatment by using Kif_BG505, related to **Fig 3c**. The threshold of peak area was reduced from 5E8 to 5E7.

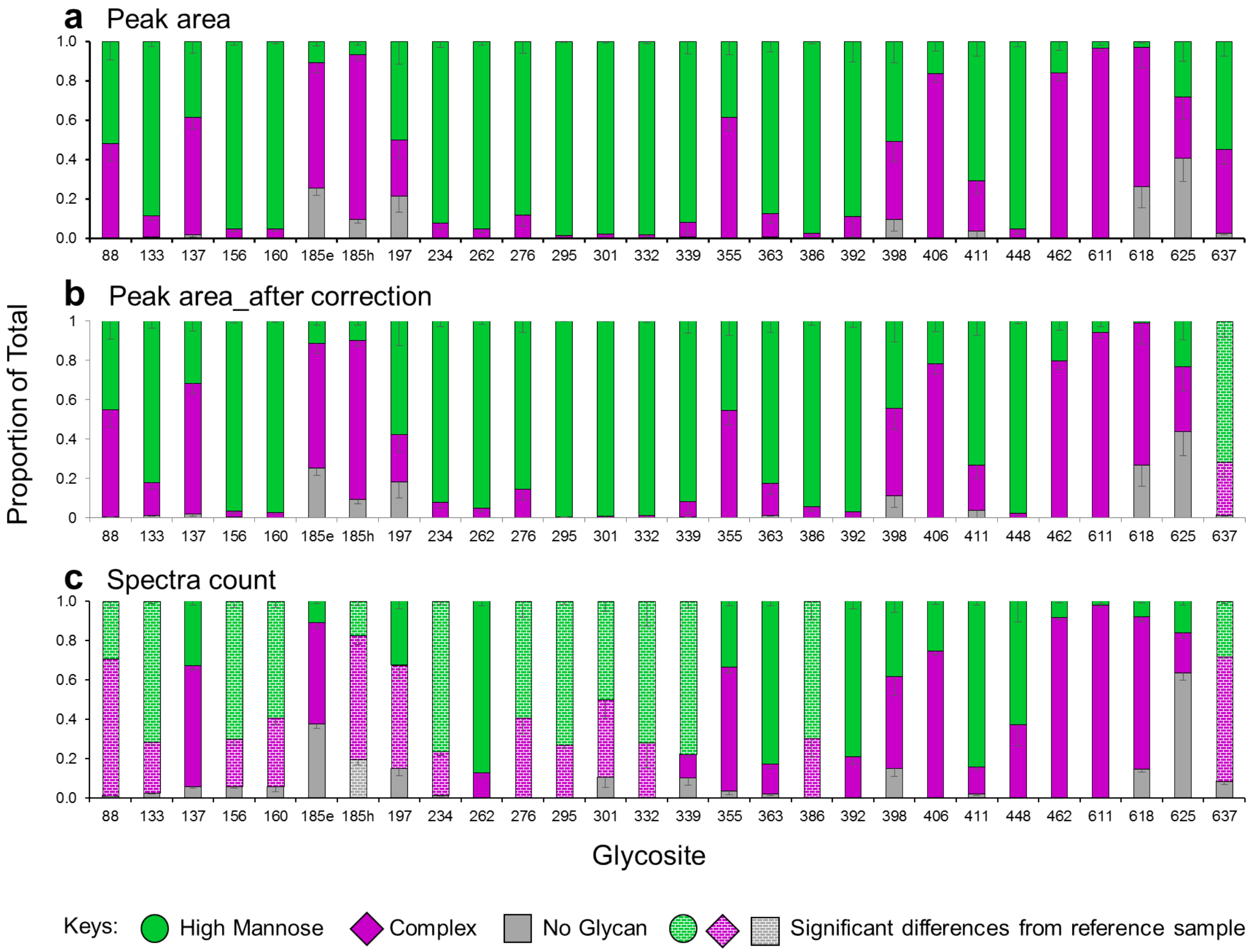

Supplementary Figure 6

Site-specific glycosylation of BG505 SOSIP trimer purified with Ni^{2+}/SEC: (a) reference dataset based on peak area, as shown also in Fig 1c & 1d; (b) after correcting peak area with site specific ratio of high-mannose and complex-type glycans determined from data Fig. 3d; (c) based on spectra counts.

```
                            Signal Sequence
                            1        10        20        30        40
HXB2                        MRVKEK..YQHLWRWGWRWGT...MLLGMLMICSATEKLWVTVYYGVPVWKEA
BG505 SOSIP.664 trimer      .................................TGAENLWVTVYYGVPVWKDA
BG505 monomer               .................................TG.............VWKDA
JR-FL SOSIP.664             ...................................VEKLWVTVYYGVPVWKEA
B41 SOSIP.664               MDAMKRGLCCVLLLCGAVFVSPSQEIHARFRRGARAAKKWVTVYYGVPVWKEA
CRF02_AG_250 SOSIP.664      ...................................AEKLWVTVYYGVPVWREA
327c SOSIP.664              MDAMKRGLCCVLLLCGAVFVSPSQEIHARFRRGARMGNLWVTVYYGVPVWKEA
SIVcpzMT145 SOSIP.664       ....................................ENWWVTVYYGVPVWREA

                            C1
                            50        60        70        80        90        100
HXB2                        TTTLFCASDAKAYDTEVHNVWATHACVPTDPNPQEVVLVNVTENFNMWKNDMV
BG505 SOSIP.664 trimer      ETTLFCASDAKAYETEKHNVWATHACVPTDPNPQEIHLENVTEEFNMWKNNMV
BG505 monomer               ETTLFCASDAKAYETEKHNVWATHACVPTDPNPQEIHLENVTEEFNMWKNNMV
JR-FL SOSIP.664             TTTLFCASDAKAYDTEVHNVWATHACVPTDPNPQEVVLENVTEHFNMWKNNMV
B41 SOSIP.664               TTTLFCASDAKAYDTEVHNVWATHACVPTDPNPQEIVLGNVTENFNMWKNNMV
CRF02_AG_250 SOSIP.664      DTTLFCASDAKGYDTEAHNVWATHACVPTDPRPQEMYLENVTENFNMWKNSMV
327c SOSIP.664              KTTLFCASDAKAYEQEVHNVWATHACVPTDPNPQEMRMENVTENFNMWKNDMV
SIVcpzMT145 SOSIP.664       KTTLFCASDAKSYSTEAHNIWATQACVPTDPTPQEVLLPNVTEEFNMWENYMV

                                                               V1
                               110       120       130       140       150
HXB2                        EQMHEDIISLWDQSLKPCVKLTPLCVSLKCTDLKNDTNTNSS....SGRMIME
BG505 SOSIP.664 trimer      EQMHTDIISLWDQSLKPCVKLTPLCVTLQCTNVTNNITDD..........MR.
BG505 monomer               EQMHTDIISAWDQSLKPCVKLTPLCVTLQCTNVTNNITDD..........MR.
JR-FL SOSIP.664             EQMQEDIISLWDQSLKPCVKLTPLCVTLNCKDVNATNTTNDS....EG..TME
B41 SOSIP.664               EQMHEDIISLWDQSLKPCVKLTPLCVTLNCNNVNTNNTNNSTNATISDWEKME
CRF02_AG_250 SOSIP.664      EQMHTDIISLWDESLKPCVKLTPLCVTLDCQAFNSSSHTNSS........IAM
327c SOSIP.664              DQMHEDIISLWDQSLKPCVKLTPLCVTLDCQNVNATQNTNDTIS.........
SIVcpzMT145 SOSIP.664       DQMQEDIISLWEQSLKPCVKLTPLCVTLTCNNPTNTSCTNSTDDRL.......

                                            V2
                                 160       170       180                 190
HXB2                        KGEIKNCSFNISTSIRGKVQKEYAFFYKLDIIPID........N.......DTTS
BG505 SOSIP.664 trimer      .GELKNCSFNMTTELRDKKQKVYSLFYRLDVVQINENQGNRSNN.......SNKE
BG505 monomer               .GELKNCSFNMTTELRDKKQKVYSLFYRLDVVQINENQGNRSNN.......SNKE
JR-FL SOSIP.664             RGEIKNCSFNITTSIRDKVQKEYALFYKLDVVPID........N.......NNTS
B41 SOSIP.664               TGEMKNCSFNVTTSIRDKIKKEYALFYKLDVVPLE........NKNNINNTNITN
CRF02_AG_250 SOSIP.664      Q.EMKNCSFNVTTELRDKKKKEYSFFYKTDIEQIN........K.......NGRQ
327c SOSIP.664              ..TMKNCTFNTTADLGDKKQKGRALFYNLDIVQLN........P.......NSNS
SIVcpzMT145 SOSIP.664       .GDMRNCSFNVTTELRDKKRKVYSLFYVEDITAIG........N.......NST.

                                                C2
                                  200       210       220       230       240
HXB2                        ..YKLTSCNTSVITQACPKVSFEPIPIHYCAPAGFAILKCNNKTFNGTGPCTNVS
BG505 SOSIP.664 trimer      ..YRLINCNTSAITQACPKVSFEPIPIHYCAPAGFAILKCKDKKFNGTGPCPSVS
BG505 monomer               ..YRLINCNTSAITQACPKVSFEPIPIHYCAPAGFAILKCKDKKFNGTGPCPSVS
JR-FL SOSIP.664             ..YRLISCDTSVITQACPKISFEPIPIHYCAPAGFAILKCNDKTFNGKGPCKNVS
B41 SOSIP.664               ..YRLINCNTSVITQACPKVSFEPIPIHYCAPAGFAILKCNSKTFNGSGPCTNVS
CRF02_AG_250 SOSIP.664      ..YRLINCNTSAITQACPKVSFEPIPIHFCAPAGFAILKCNEKHFNGKGPCKNVS
327c SOSIP.664              SEYRLISCNTSTITQACPKVSFEPIPIHYCAPAGYAILKCNNKTFNGLGPCTNVS
SIVcpzMT145 SOSIP.664       ..YRLINCNTTAITQACPKTSFEPIPIHYCAPAGFALLKCNDIDYKGNETCKNVS

                                250       260       270       280       290       300
HXB2                        TVQCTHGIRPVVSTQLLLNGSLAEEEVVIRSVNFTDNAKTIIVQLNTSVEINCTRPN
BG505 SOSIP.664 trimer      TVQCTHGIKPVVSTQLLLNGSLAEEEVMIRSENITNNAKNILVQFNTPVQINCTRPN
BG505 monomer               TVQCTHGIKPVVSTQLLLNGSLAEEEVMIRSENITNNAKNILVQFNTPVQINCTRPN
JR-FL SOSIP.664             TVQCTHGIRPVVSTQLLLNGSLAEEEVVIRSDNFTNNAKTIIVQLKESVEINCTRPN
B41 SOSIP.664               TVQCTHGIRPVVSTQLLLNGSLAEEEIVIRSENITDNAKTIIVQLNEAVEINCTRPN
CRF02_AG_250 SOSIP.664      TVQCTHGIKPVVSTQLLLNGSLAEEEVVIRVENTIDNAKTIIVQLAKPVKINCTRPN
327c SOSIP.664              TVQCTHGIKPVVSTQLLLNGSLAEGEIIIRSENLTDNGKTIIVHLNESVKIVCIRPN
SIVcpzMT145 SOSIP.664       TVHCTHGIKPVATTQLILNGSTADNQTVAR...IDPSENLAIIQLKDPVKITCRRPG

                                      V3                            C3
                                  310       320       330       340       350
HXB2                        NNTRKRIRIQRGPGRAFVTIGK.IGNMRQAHCNISRAKWNNTLKQIASKLREQFG
BG505 SOSIP.664 trimer      NNTRKSIRI..GPGQAFYATGDIIGDIRQAHCNVSKATWNETLGKVVKQLRKHFG
BG505 monomer               NNTRKSIRI..GPGQAFYATGDIIGDIRQAHCNVSKATWNETLGKVVKQLRKHFG
JR-FL SOSIP.664             NNTRKSIHI..GPGRAFYTTGEIIGDIRQAHCNISRAKWNDTLKQIVIKLREQFE
B41 SOSIP.664               NNTRKSIHI..GPGRAFYATGDIIGNIRQAHCNISKARWNETLGQIVAKLEEQFP
CRF02_AG_250 SOSIP.664      NNTRKSIRI..GPGQTFYATGDIIGNIRKAYCNVSKREWNNTLQQVAAQLSKSFN
327c SOSIP.664              NNTRKSIRI..GPGQTFFAT.DIIGDIRQAYCNISREDWNKTLDRVRKKLEEHFP
SIVcpzMT145 SOSIP.664       NNTRGQIQI..GPAMTFYNIENVVGDTRKAYCEINGTQWAKALNETKEVLRN‾LR

                                                           V4
                               360       370       380       390       400
HXB2                        NNKTIIFKQSSGGDPEIVTHSFNCGGEFFYCNSTQLFNSTWFNSTW..S..TE
BG505 SOSIP.664 trimer      NNTIIRFANSSGGDLEVTTHSFNCGGEFFYCNTSGLFNSTWISNT...S..VQ
BG505 monomer               NNTIIRFANSSGGDLEVTTHSFNCGGEFFYCNTSGLFNSTWISNT...S..VQ
JR-FL SOSIP.664             N.KTIVFNHSSGGDPEIVMHSFNCGGEFFYCNSTQLFNSTWNN.....N..TE
B41 SOSIP.664               N.KTIIFNHSSGGDPEIVTHSFNCGGEFFYCNTTPLFNSTWNN.....T..RT
CRF02_AG_250 SOSIP.664      NTK.IVFEKHSGGDLEVITHSFVCGGEFFYCNTSGLFNSTWHNSTW..TNSTT
327c SOSIP.664              N.KTIEFKRHSGGDLEVTQHSFNCRGEFFYCNTTHLFENTTYTN.........
SIVcpzMT145 SOSIP.664       KN..ISFMVPSGGDPEVTNHHFNCGGEFFYCNTSEIINITKIN.........K

                                                  C4
                                410       420       430       440       450
HXB2                        GSNNTEGSDTITLPCRIKQIINMWQKVGKAMYAPPISGQIRCSSNITGLLL
BG505 SOSIP.664 trimer      GSNSTGSNDSITLPCRIKQIINMWQRIGQAMYAPPIQGVIRCVSNITGLIL
BG505 monomer               GSNSTGSNDSITLPCRIKQIINMWQRIGQAMYAPPIQGVIRCVSNITGLIL
JR-FL SOSIP.664             GSNNTEG.NTITLPCRIKQIINMWQEVGKAMYAPPIRGQIRCSSNITGLLL
B41 SOSIP.664               DDYPTGGEQNITLQCRIKQIINMWQGVGKAMYAPPIRGQIRCSSNITGLLL
CRF02_AG_250 SOSIP.664      GSNGTESNDTITLQCEIKQFINMWQRVGRAMYAPPIPGVIRCESDITGLLL
327c SOSIP.664              .......SSNITLPCRIKQIINMWQGVGRAMYAPPIAGNITCISNITGILL
SIVcpzMT145 SOSIP.664       TENMT......IIPCRIRQIVNSWMRVGKGIFAPPIRGNITCTSNITGMLL
```

```
                                                V5                 C5
                                  460       470       480       490       500
HXB2                        TR.....DGGNSKNESEIFR.PGGGDMRDNWRSELYKYKVVKIEPLGVAPTK
BG505 SOSIP.664 trimer      TR.....DGGSTNSTTETFR.PGGGDMRDNWRSELYKYKVVKIEPLGVAPTR
BG505 monomer               TR.....DGGSTNSTTETFR.PGGGDMRDNWRSELYKYKVVKIEP.......
JR-FL SOSIP.664             TR.....DGGINENGTEIFR.PGGGDMRDNWRSELYKYKVVKIEPLGVAPTK
B41 SOSIP.664               TR.....DGGRDQNGTETFR.PGGGNMRDNWRSELYKYKVVKIEPLGIAPTA
CRF02_AG_250 SOSIP.664      TR.....DGPNS.TQNETFR.PGGGDMRDNWRSELYKYKVVQIEPLGVAPTH
327c SOSIP.664              TR.....DGGNN.GTNETFR.PGGGDMRDNWRSELYKYKVVEIKPLGIAPTK
SIVcpzMT145 SOSIP.664       EIHKNREDQGEDQDQNNTYVCLTGGNMKDIWRSELYKYKIVEIQPLGVAPTK

                                       510       520       530       540
HXB2                        AKRRVV......QREKRAVGIGALFLGFLGAAGSTMGAASMTLTVQARQLL
BG505 SOSIP.664 trimer      CKRRVVG....RRRRRRAVGIGAVFLGFLGAAGSTMGAASMTLTVQARNLL
BG505 monomer               ...................................................
JR-FL SOSIP.664             CKRRVR......RRKKRAVGIGAVFLGFLGAAGSTMGAASMTLTVQARLLL
B41 SOSIP.664               CKRRVVQ....RRRRRRAVGLGAFILGFLGAAGSTMGAASMALTVQARLLL
CRF02_AG_250 SOSIP.664      CKRRVVE....RRRRRRAVGLGAVFFGFLGAAGSTMGAASITLTVQARQLL
327c SOSIP.664              CKRRVVE....RRRRRRAVGMGALFLGFLGAAGSTMGAASMTLTVQARQLL
SIVcpzMT145 SOSIP.664       CRRYAVEKQHHRRRRRRALGLGALFLGFLGAAGSTMGAASVVLTVQARQLL

                               550       560       570       580       590
HXB2                        SGIVQQQNNLLRAIEAQQHLLQLTVWGIKQLQAGILAVERYLKDQQLLGIW
BG505 SOSIP.664 trimer      SGIVQQQSNLLRAPEAQQHLLKLTVWGIKQLQARVLAVERYLRDQQLLGIW
BG505 monomer               ...................................................
JR-FL SOSIP.664             SGIVQQQNNLLRAPEAQQRMLQLTVWGIKQLQARVLAVERYLGDQQLLGIW
B41 SOSIP.664               SGIVQQQNNLLRAPEAQQHMLQLTVWGIKQLQARVLAVERYLRDQQLLGIW
CRF02_AG_250 SOSIP.664      SGIVQQQSNWLKAPEAQQHLLRLTVWGIKQLQARVLALERYLKDQQLLGIW
327c SOSIP.664              SGIVQQQSNLLRAPEAQQHMLQLTVWGIKQLQARVLALERYLQDQQLLGIW
SIVcpzMT145 SOSIP.664       TGIVQQQNNLLRAPEAQQHLLQLSVWGIKQLQARVLAVERYLRDQQLLGLW

                              600       610       620       630       640
HXB2                        GCSGRLICTTAVPWNASWSNK.SLEQIWNHTTWMEWDREINNYTSLIHSLI
BG505 SOSIP.664 trimer      GCSGKLICCTNVPWNSSWSNR.NLSEIWDNMTWLQWDKEISNYTQIIYGLL
BG505 monomer               ...................................................
JR-FL SOSIP.664             GCSGKLICCTAVPWNASWSNK.SLDRIWNNMTWMEWEREIDNYTSEIYTLI
B41 SOSIP.664               GCSGKIICCTNVPWNDSWSNK.TINEIWDNMTWMQWEKEIDNYTQHIYTLL
CRF02_AG_250 SOSIP.664      GCSGKLICCTTVPWNSSWSNK.NYTDIWDNMTWLQWDREISNYTDEIYRLI
327c SOSIP.664              GCSGKLICCTAVPWNSSWSNK.TYNDIWDNMTWMQWEREISNYTNTIYTLL
SIVcpzMT145 SOSIP.664       GCTGKTICCTAVRWNKTWGNISDYQVIWNNYTWQQWDREVNNYTGLIYTLL

                              650       660       670       680       690
HXB2                        EESQNQHEKNEQELLELDKWASLWNWFNITNWLWYTKLFIMIVGGLVGLR
BG505 SOSIP.664 trimer      EESQNQQEKNEQDLLALD................................
BG505 monomer               ..................................................
JR-FL SOSIP.664             EESQNQQEKNEQELLEL..............................G..
B41 SOSIP.664               EVSQIQQEKNEQELLELD.............................G..
CRF02_AG_250 SOSIP.664      EQSQNQQEKNEQDLLALD.............................G..
327c SOSIP.664              EVSQNQQEQNEKDLLALD.............................G..
SIVcpzMT145 SOSIP.664       EEANTQQEKNEKELLELD.............................G..

                              700       710       720       730       740
HXB2                        IVFAVLSIVNRVRQGYSPLSFQTHLPTPRGPDRPEGIEEEGGERDRDRSI
BG505 SOSIP.664 trimer      ..................................................
BG505 monomer               ..................................................
JR-FL SOSIP.664             ........................................GG........
B41 SOSIP.664               .......................................SG.........
CRF02_AG_250 SOSIP.664      .......................................SGS........
327c SOSIP.664              .......................................SG.........
SIVcpzMT145 SOSIP.664       .......................................SGS........

                              750       760       770       780       790
HXB2                        RLVNGSLALIWDDLRSLCLFSHHRLRDLLLIVTRIVELLGRRGWEALKYWWN
BG505 SOSIP.664 trimer      .............................................GTKHHHH
BG505 monomer               .............................................GTKHHHH
JR-FL SOSIP.664             ....G................HHHHHH.........................
B41 SOSIP.664               ........................LNDIF...............EAQKIEWH
CRF02_AG_250 SOSIP.664      ............................................GGSGHHHH
327c SOSIP.664              ........................LNDIF...............EAQKIEWH
SIVcpzMT145 SOSIP.664       ............................................GGSGHHHH

                            800       810       820       830       840       850
HXB2                        LLQYWSQELKNSAVSLLNATAIAVAEGTDRVIEVVQGACRAIRHIPRRIRQGLERILL
BG505 SOSIP.664 trimer      HH........................................................
BG505 monomer               HH........................................................
JR-FL SOSIP.664             ..........................................................
B41 SOSIP.664               E.........................................................
CRF02_AG_250 SOSIP.664      HHHH......................................................
327c SOSIP.664              E.........................................................
SIVcpzMT145 SOSIP.664       HHHH......................................................
```

Supplementary Figure 7

Amino acid sequences of Envs analyzed in this study.
Numbering positions were aligned with the reference HXB2 sequence by using Clustal W Protein sequences. Potential N-glycosites are highlighted in red, while locations of conserved (C1 to C5) and variable (V1 to V5) regions were indicated by using blue and magenta lines above sequences respectively.

Supplementary Table 1
The number of MS/MS spectra that could be detected from each glycosylation site of bovine fetuin.

Glycosite	N+0	N+3	N+203	Sum
99	1	565	3	569
156	2	218	2	222
176	75	624	0	699

Supplementary Table 2

The number of MS/MS spectra that could be detected from each glycosylation site of the recombinant influenza virus hemagglutinin from strain A/Victoria/361/2011 produced in HEK 293 F cells.

Glycosite	N+0	N+3	N+203	Sum
22	0	450	11	461
38	0	451	86	537
45	80	66	28	174
63	9	318	3	330
122	298	1	5	304
126	9	327	92	428
133	20	118	193	331
144	47	36	2	85
165	0	197	626	823
246	17	66	185	268
285	17	83	415	515
483	1	295	5	301

Supplementary Table 3
The number of MS/MS spectra that could be detected from each glycosylation site of BG505 SOSIP.664 trimer, related to **Fig. 1d**.

Glycosite	N+0	N+3	N+203	Sum
88	20	965	448	1433
133	36	403	1076	1515
137	76	939	534	1549
156	37	167	395	599
160	9	213	304	526
185e	662	778	193	1633
185h	275	789	218	1282
197	149	292	259	700
234	16	215	802	1033
262	2	148	1262	1412
276	0	208	435	643
295	0	58	157	215
301	5	44	41	90
332	0	32	214	246
339	46	74	451	571
355	43	741	414	1198
363	28	194	881	1103
386	0	92	204	296
392	0	38	133	171
398	82	214	208	504
406	0	585	189	774
411	17	121	661	799
448	0	37	62	99
462	0	2138	179	2317
611	0	164	9	173
618	159	815	88	1062
625	694	209	166	1069
637	35	273	118	426

Supplementary Table 4
Site-specific glycosylation of BG505 SOSIP.664 trimer purified over Ni^{2+}/SEC, related to **Fig. 1d**.

Glycosite	N+0	N+3	N+203	Standard Error (N+0)	Standard Error (N+3)	Standard Error (N+203)
88	0.0034	0.4792	0.5174	0.0014	0.0904	0.0898
133	0.0071	0.1081	0.8848	0.0024	0.0241	0.0255
137	0.0166	0.5988	0.3846	0.0082	0.0605	0.0593
156	0.0042	0.0416	0.9542	0.0024	0.0148	0.0154
160	0.0001	0.0459	0.9540	0.0001	0.0092	0.0092
185e	0.2555	0.6392	0.1053	0.0374	0.0523	0.0201
185h	0.0968	0.8362	0.0669	0.0205	0.0285	0.0157
197	0.2128	0.2866	0.5006	0.0804	0.0911	0.1152
234	0.0014	0.0755	0.9231	0.0005	0.0298	0.0297
262	0.0000	0.0483	0.9517	0.0000	0.0184	0.0184
276	0.0000	0.1188	0.8812	0.0000	0.0565	0.0565
295	0.0000	0.0138	0.9862	0.0000	0.0025	0.0025
301	0.0021	0.0201	0.9778	0.0012	0.0065	0.0058
332	0.0000	0.0172	0.9828	0.0000	0.0097	0.0097
339	0.0058	0.0751	0.9191	0.0034	0.0590	0.0601
355	0.0028	0.6136	0.3836	0.0012	0.0669	0.0668
363	0.0071	0.1183	0.8746	0.0027	0.0478	0.0502
386	0.0000	0.0234	0.9766	0.0000	0.0087	0.0087
392	0.0000	0.1105	0.8895	0.0000	0.1041	0.1041
398	0.0966	0.3958	0.5076	0.0613	0.1036	0.1063
406	0.0000	0.8374	0.1626	0.0000	0.0452	0.0452
411	0.0379	0.2555	0.7065	0.0356	0.0691	0.0734
448	0.0000	0.0469	0.9531	0.0000	0.0247	0.0247
462	0.0000	0.8433	0.1567	0.0000	0.0441	0.0441
611	0.0000	0.9683	0.0317	0.0000	0.0169	0.0169
618	0.2617	0.7100	0.0283	0.1047	0.1053	0.0075
625	0.4080	0.3112	0.2808	0.1210	0.1150	0.1000
637	0.0268	0.4256	0.5476	0.0079	0.0743	0.0728

Supplementary Table 5

The number of MS/MS spectra that could be detected from each glycosylation site of Kif_BG505 treated with Endo H followed by PNGase F deglycosylation, related to **Fig. 3b**. Peptides that had potential glycosites, but were not glycosylated were not included.

Glycosite	N+3	N+203	Sum
88	80	925	1005
133	152	703	855
137	116	705	821
156	40	221	261
160	13	210	223
185e	78	403	481
185h	21	278	299
197	32	274	306
234	26	782	808
262	33	2092	2125
276	57	239	296
295	16	123	139
301	3	37	40
332	30	198	228
339	30	301	331
355	50	265	315
363	56	456	512
386	29	133	162
392	2	52	54
398	4	215	219
406	38	376	414
411	23	395	418
448	13	48	61
462	96	629	725
611	14	63	77
618	44	759	803
625	4	357	361
637	6	82	88

Supplementary Table 6

The number of MS/MS spectra that could be detected from each glycosylation site of Kif_BG505 treated with PNGase F only, related to **Fig. 3c**. Peptides that had potential glycosites, but were not glycosylated were not included.

Glycosite	N+3	N+203	Sum
88	1831	9	1840
133	4769	5	4774
137	4738	14	4752
156	2002	5	2007
160	884	0	884
185e	624	7	631
185h	688	10	698
197	380	3	383
234	1204	10	1214
262	4396	36	4432
276	870	0	870
295	559	0	559
301	378	0	378
332	809	1	810
339	799	3	802
355	1022	0	1022
363	3844	20	3864
386	84	0	84
392	41	0	41
398	105	8	113
406	692	6	698
411	799	0	799
448	131	0	131
462	1494	19	1513
611	110	0	110
618	500	15	515
625	256	0	256
637	201	0	201

Supplementary Table 7

The number of MS/MS spectra that could be detected from the digestion mixture of Kif_BG505 containing homogeneous N+3 and N+203 modifications at a molar ratio of 1:1, related to **Fig. 3d**. Peptides that had potential glycosites, but were not glycosylated were not included.

Glycosite	N+3	N+203	Sum
88	1508	1065	2573
133	3991	599	4590
137	3901	637	4538
156	2118	270	2388
160	532	174	706
185e	463	316	779
185h	446	157	603
197	330	220	550
234	899	672	1571
262	2758	1755	4513
276	901	233	1134
295	369	96	465
301	246	21	267
332	583	219	802
339	567	310	877
355	839	182	1021
363	2095	414	2509
386	120	44	164
392	23	6	29
398	47	146	193
406	486	247	733
411	560	274	834
448	110	23	133
462	797	424	1221
611	109	71	180
618	582	825	1407
625	357	332	689
637	193	71	264

Supplementary Table 8
The number of MS/MS spectra that could be detected from each glycosylation site of BG505 SOSIP.664 trimer from second biological batch purified by Ni^{2+}/SEC, related to **Fig. 4a**.

Glycosite	N+0	N+3	N+203	Sum
88	24	992	315	1331
133	25	536	1137	1698
137	81	1097	547	1725
156	35	221	338	594
160	8	252	213	473
185e	682	831	146	1659
185h	212	766	122	1100
197	135	270	238	643
234	14	323	1038	1375
262	0	128	1203	1331
276	0	169	244	413
295	0	39	114	153
301	6	13	17	36
332	1	188	267	456
339	116	73	337	526
355	33	782	243	1058
363	45	187	706	938
386	0	76	77	153
392	0	18	82	100
398	110	178	197	485
406	0	523	101	624
411	8	105	536	649
448	0	20	47	67
462	0	1162	112	1274
611	0	65	2	67
618	93	671	50	814
625	515	122	194	831
637	78	243	58	379

Supplementary Table 9

Site-specific glycosylation of BG505 SOSIP.664 trimer from second biological batch purified by Ni^{2+}/SEC, related to **Fig. 4a**. The proportions of no glycan, complex type, and high mannose glycans that showed significant differences when compared to the glycosylation of BG505 SOSIP.664 trimer purified by Ni^{2+}/SEC shown in **Fig. 1d** were highlighted in purple.

Glycosite	N+0	N+3	N+203	Standard Error (N+0)	Standard Error (N+3)	Standard Error (N+203)
88	0.0031	0.5759	0.4210	0.0015	0.0895	0.0891
133	0.0040	0.1669	0.8291	0.0016	0.0489	0.0490
137	0.0180	0.4528	0.5292	0.0084	0.0516	0.0512
156	0.0012	0.2243	0.7744	0.0007	0.0873	0.0871
160	0.0162	0.2100	0.7739	0.0129	0.0801	0.0880
185e	0.1854	0.7215	0.0931	0.0432	0.0558	0.0237
185h	0.0805	0.8469	0.0725	0.0260	0.0465	0.0305
197	0.2008	0.3741	0.4251	0.0966	0.1081	0.1133
234	0.0003	0.1120	0.8877	0.0003	0.0337	0.0337
262	0.0000	0.0413	0.9587	0.0000	0.0150	0.0150
276	0.0000	0.1189	0.8811	0.0000	0.0601	0.0601
295	0.0000	0.0263	0.9737	0.0000	0.0048	0.0048
301	0.0021	0.0204	0.9775	0.0015	0.0091	0.0078
332	0.0001	0.0513	0.9485	0.0001	0.0347	0.0347
339	0.0530	0.2300	0.7170	0.0487	0.0972	0.1039
355	0.0026	0.6416	0.3558	0.0013	0.0685	0.0688
363	0.0028	0.1257	0.8715	0.0016	0.0561	0.0558
386	0.0000	0.1913	0.8087	0.0000	0.1003	0.1003
392	0.0000	0.0000	1.0000	0.0000	0.0000	0.0000
398	0.0824	0.4486	0.4690	0.0439	0.0923	0.0864
406	0.0000	0.8093	0.1907	0.0000	0.0361	0.0361
411	0.0006	0.1427	0.8567	0.0003	0.0518	0.0517
448	0.0000	0.0099	0.9901	0.0000	0.0099	0.0099
462	0.0000	0.8551	0.1449	0.0000	0.0509	0.0509
611	0.0000	1.0000	0.0000	0.0000	0.0000	0.0000
618	0.2636	0.7251	0.0113	0.1067	0.1055	0.0037
625	0.5886	0.0432	0.3682	0.1219	0.0134	0.1119
637	0.0410	0.5979	0.3611	0.0109	0.0762	0.0776

Supplementary Table 10

The number of MS/MS spectra that could be detected from each glycosylation site of BG505 SOSIP.664 trimer purified over 2G12/SEC, related to **Fig. 4b**.

Glycosite	N+0	N+3	N+203	Sum
88	20	1075	293	1388
133	19	500	786	1305
137	55	891	386	1332
156	18	238	265	521
160	0	185	98	283
185e	552	1061	137	1750
185h	308	924	199	1431
197	100	268	257	625
234	0	255	733	988
262	2	347	1876	2225
276	13	147	191	351
295	0	38	109	147
301	5	12	26	43
332	8	146	197	351
339	112	74	222	408
355	25	538	132	695
363	56	288	919	1263
386	0	97	75	172
392	0	22	30	52
398	59	139	154	352
406	0	416	98	514
411	4	93	429	526
448	0	19	19	38
462	0	1149	118	1267
611	2	81	106	189
618	117	552	41	710
625	356	238	139	733
637	31	126	26	183

Supplementary Table 11

Site-specific glycosylation of BG505 SOSIP.664 trimer purified over 2G12/SEC, related to **Fig. 4b**. The proportions of no glycan, complex type, and high mannose glycans that showed significant differences when compared to the glycosylation of BG505 SOSIP.664 trimer purified by Ni^{2+}/SEC shown in **Fig. 1d** were highlighted in purple.

Glycosite	N+0	N+3	N+203	Standard Error (N+0)	Standard Error (N+3)	Standard Error (N+203)
88	0.0013	0.6460	0.3527	0.0007	0.0744	0.0739
133	0.0018	0.1217	0.8765	0.0008	0.0532	0.0538
137	0.0239	0.3698	0.6063	0.0141	0.0880	0.0869
156	0.0028	0.0704	0.9268	0.0019	0.0336	0.0335
160	0.0000	0.0734	0.9266	0.0000	0.0267	0.0267
185e	0.2022	0.7352	0.0626	0.0416	0.0525	0.0131
185h	0.1100	0.7630	0.1270	0.0366	0.0644	0.0475
197	0.1889	0.4100	0.4011	0.1448	0.1177	0.1152
234	0.0000	0.0485	0.9515	0.0000	0.0104	0.0104
262	0.0001	0.0750	0.9249	0.0001	0.0403	0.0404
276	0.0003	0.0739	0.9258	0.0003	0.0208	0.0208
295	0.0000	0.0188	0.9812	0.0000	0.0083	0.0083
301	0.0007	0.0007	0.9986	0.0007	0.0007	0.0014
332	0.0000	0.0664	0.9336	0.0000	0.0375	0.0375
339	0.0505	0.0499	0.8996	0.0286	0.0238	0.0357
355	0.0071	0.4851	0.5078	0.0053	0.1347	0.1361
363	0.0015	0.0580	0.9405	0.0009	0.0276	0.0284
386	0.0000	0.2050	0.7950	0.0000	0.1067	0.1067
392	0.0000	0.2500	0.7500	0.0000	0.1247	0.1247
398	0.0895	0.4117	0.4988	0.0571	0.1027	0.0817
406	0.0000	0.8439	0.1561	0.0000	0.0354	0.0354
411	0.0003	0.0682	0.9316	0.0001	0.0474	0.0475
448	0.0000	0.7000	0.3000	0.0000	0.1856	0.1856
462	0.0000	0.8744	0.1256	0.0000	0.0724	0.0724
611	0.0000	0.9924	0.0076	0.0000	0.0076	0.0076
618	0.2990	0.6446	0.0563	0.2338	0.2205	0.0492
625	0.2766	0.3065	0.4169	0.2429	0.2456	0.0027
637	0.0281	0.5889	0.3830	0.0136	0.1364	0.1399

Supplementary Table 12

The number of MS/MS spectra hits that could be detected from each glycosylation site of BG505 SOSIP.664 trimer purified over PGT145/SEC, related to **Fig. 4b**.

Glycosite	N+0	N+3	N+203	Sum
88	17	1535	316	1868
133	21	211	1154	1386
137	47	842	533	1422
156	0	56	362	418
160	7	9	45	61
185e	571	1010	166	1747
185h	246	1208	323	1777
197	154	418	391	963
234	13	48	970	1031
262	3	29	1549	1581
276	16	110	392	518
295	0	23	138	161
301	3	2	15	20
332	40	120	426	586
339	121	49	279	449
355	68	544	192	804
363	32	240	1102	1374
386	2	69	168	239
392	0	11	36	47
398	98	118	236	452
406	0	589	94	683
411	0	69	633	702
448	0	16	70	86
462	0	1835	102	1937
611	0	62	0	62
618	97	701	52	850
625	380	234	264	878
637	34	212	52	298

Supplementary Table 13
Site-specific glycosylation of BG505 SOSIP.664 trimer purified over PGT145/SEC, related to **Fig. 4b**. The proportions of no glycan, complex type, and high mannose glycans that showed significant differences when compared to the glycosylation of BG505 SOSIP.664 trimer purified by Ni^{2+}/SEC shown in **Fig. 1d** were highlighted in purple.

Glycosite	N+0	N+3	N+203	Standard Error (N+0)	Standard Error (N+3)	Standard Error (N+203)
88	0.0007	0.6545	0.3448	0.0003	0.0729	0.0726
133	0.0038	0.0494	0.9469	0.0017	0.0204	0.0220
137	0.0563	0.6417	0.3020	0.0325	0.0605	0.0594
156	0.0000	0.0210	0.9790	0.0000	0.0155	0.0155
160	0.0000	0.0000	1.0000	0.0000	0.0000	0.0000
185e	0.1796	0.7448	0.0757	0.0464	0.0551	0.0312
185h	0.1156	0.7836	0.1008	0.0334	0.0521	0.0369
197	0.0394	0.5981	0.3625	0.0083	0.0703	0.0705
234	0.0001	0.0070	0.9929	0.0001	0.0025	0.0025
262	0.0002	0.0127	0.9871	0.0001	0.0069	0.0070
276	0.0006	0.1981	0.8013	0.0006	0.0775	0.0775
295	0.0000	0.0067	0.9933	0.0000	0.0039	0.0039
301	0.0054	0.0000	0.9946	0.0008	0.0000	0.0008
332	0.0000	0.0859	0.9141	0.0000	0.0478	0.0478
339	0.0209	0.0064	0.9726	0.0150	0.0026	0.0152
355	0.0083	0.6023	0.3893	0.0040	0.0578	0.0580
363	0.0078	0.0067	0.9855	0.0070	0.0025	0.0071
386	0.0000	0.1007	0.8993	0.0000	0.0518	0.0518
392	0.0000	0.3416	0.6584	0.0000	0.1712	0.1712
398	0.0403	0.5152	0.4445	0.0077	0.0856	0.0807
406	0.0000	0.8522	0.1478	0.0000	0.0367	0.0367
411	0.0000	0.0357	0.9643	0.0000	0.0178	0.0178
448	0.0000	0.0118	0.9882	0.0000	0.0000	0.0000
462	0.0000	0.8820	0.1180	0.0000	0.0676	0.0676
611	0.0000	1.0000	0.0000	0.0000	0.0000	0.0000
618	0.3617	0.6095	0.0288	0.1395	0.1369	0.0116
625	0.3796	0.2822	0.3382	0.1604	0.0906	0.0999
637	0.0122	0.5231	0.4647	0.0073	0.0979	0.0960

Supplementary Table 14

The number of MS/MS spectra that could be detected from each glycosylation site of BG505 SOSIP.664 trimer purified over PGT151/SEC, related to **Fig. 4b**.

Glycosite	N+0	N+3	N+203	Sum
88	23	1625	554	2202
133	34	265	1544	1843
137	71	1131	694	1896
156	11	68	472	551
160	18	94	269	381
185e	450	945	134	1529
185h	218	718	114	1050
197	187	343	353	883
234	1	46	1080	1127
262	1	27	1677	1705
276	15	82	264	361
295	0	21	332	353
301	1	2	18	21
332	10	85	247	342
339	131	45	353	529
355	47	529	276	852
363	25	303	1163	1491
386	0	81	125	206
392	0	11	94	105
398	92	399	220	711
406	0	948	165	1113
411	8	131	1008	1147
448	0	7	32	39
462	0	2108	128	2236
611	0	102	0	102
618	141	1371	21	1533
625	949	309	335	1593
637	42	267	70	379

Supplementary Table 15

Site-specific glycosylation of BG505 SOSIP.664 trimer purified over PGT151/SEC, related to **Fig. 4b**. The proportions of no glycan, complex type, and high mannose glycans that showed significant differences when compared to the glycosylation of BG505 SOSIP.664 trimer purified by Ni^{2+}/SEC shown in **Fig. 1d** were highlighted in purple.

Glycosite	N+0	N+3	N+203	Standard Error (N+0)	Standard Error (N+3)	Standard Error (N+203)
88	0.0022	0.5860	0.4119	0.0017	0.0557	0.0557
133	0.0062	0.1100	0.8838	0.0044	0.0328	0.0340
137	0.0257	0.6145	0.3598	0.0109	0.0568	0.0562
156	0.0012	0.0470	0.9517	0.0007	0.0349	0.0349
160	0.0000	0.0702	0.9298	0.0000	0.0459	0.0459
185e	0.1296	0.7878	0.0826	0.0295	0.0407	0.0307
185h	0.0973	0.8649	0.0378	0.0320	0.0376	0.0141
197	0.1164	0.4433	0.4403	0.0564	0.0703	0.0662
234	0.0000	0.0338	0.9662	0.0000	0.0299	0.0299
262	0.0000	0.0066	0.9934	0.0000	0.0028	0.0028
276	0.0000	0.0919	0.9081	0.0000	0.0652	0.0652
295	0.0000	0.0050	0.9950	0.0000	0.0022	0.0022
301	0.0000	0.0000	1.0000	0.0000	0.0000	0.0000
332	0.0833	0.0469	0.8698	0.0833	0.0402	0.0886
339	0.0166	0.0605	0.9229	0.0091	0.0587	0.0585
355	0.0037	0.6625	0.3338	0.0024	0.0706	0.0706
363	0.0010	0.0144	0.9846	0.0005	0.0065	0.0065
386	0.0000	0.1194	0.8806	0.0000	0.0407	0.0407
392	0.0000	0.0865	0.9135	0.0000	0.0213	0.0213
398	0.0242	0.6819	0.2939	0.0056	0.0676	0.0635
406	0.0000	0.8502	0.1498	0.0000	0.0263	0.0263
411	0.0001	0.1053	0.8946	0.0000	0.0410	0.0410
448	0.0000	0.0000	1.0000	0.0000	0.0000	0.0000
462	0.0000	0.9151	0.0849	0.0000	0.0398	0.0398
611	0.0000	1.0000	0.0000	0.0000	0.0000	0.0000
618	0.2435	0.7449	0.0117	0.1009	0.1003	0.0099
625	0.5410	0.1681	0.2910	0.1078	0.0557	0.0956
637	0.0237	0.6576	0.3187	0.0081	0.0808	0.0834

Supplementary Table 16

The number of MS/MS spectra that could be detected from each glycosylation site of BG505 mutant (N276D), related to **Fig. 4c**.

Glycosite	N+0	N+3	N+203	Sum
88	36	2919	906	3861
133	63	623	1538	2224
137	110	1543	634	2287
156	47	313	534	894
160	13	255	305	573
185e	697	1013	193	1903
185h	277	970	160	1407
197	162	396	304	862
234	19	245	1063	1327
262	4	226	1755	1985
295	0	67	197	264
301	11	75	52	138
332	1	88	372	461
339	80	108	582	770
355	26	1312	404	1742
363	26	213	889	1128
386	0	82	167	249
392	1	51	94	146
398	101	352	204	657
406	0	872	177	1049
411	20	170	885	1075
448	0	15	64	79
462	0	2257	112	2369
611	2	172	5	179
618	220	1187	71	1478
625	693	489	278	1460
637	43	570	105	718

Supplementary Table 17

Site-specific glycosylation of BG505 mutant (N276D), related to **Fig. 4c**. The proportions of no glycan, complex type, and high mannose glycans that showed significant differences when compared to the glycosylation of BG505 SOSIP.664 trimer purified by Ni^{2+}/SEC shown in **Fig. 1d** were highlighted in purple.

Glycosite	N+0	N+3	N+203	Standard Error (N+0)	Standard Error (N+3)	Standard Error (N+203)
88	0.0027	0.5025	0.4948	0.0011	0.0730	0.0726
133	0.0123	0.1888	0.7989	0.0041	0.0450	0.0466
137	0.0332	0.5514	0.4154	0.0099	0.0552	0.0550
156	0.0044	0.1452	0.8503	0.0018	0.0699	0.0695
160	0.0005	0.1722	0.8272	0.0005	0.0762	0.0765
185e	0.2112	0.7087	0.0801	0.0305	0.0414	0.0164
185h	0.1259	0.8085	0.0656	0.0258	0.0369	0.0168
197	0.1081	0.5215	0.3704	0.0648	0.0956	0.0903
234	0.0007	0.1050	0.8943	0.0003	0.0286	0.0285
262	0.0001	0.1345	0.8654	0.0001	0.0506	0.0506
276	0.0000	0.0000	0.0000	0.0000	0.0000	0.0000
295	0.0000	0.0198	0.9802	0.0000	0.0050	0.0050
301	0.0022	0.0575	0.9403	0.0014	0.0246	0.0241
332	0.0000	0.0990	0.9010	0.0000	0.0698	0.0698
339	0.0235	0.1502	0.8262	0.0146	0.0637	0.0697
355	0.0014	0.7352	0.2635	0.0006	0.0569	0.0567
363	0.0094	0.0617	0.9289	0.0041	0.0334	0.0339
386	0.0000	0.0709	0.9291	0.0000	0.0307	0.0307
392	0.0000	0.0350	0.9650	0.0000	0.0350	0.0350
398	0.1034	0.5555	0.3412	0.0535	0.0951	0.0847
406	0.0000	0.7096	0.2904	0.0000	0.0601	0.0601
411	0.0040	0.1381	0.8579	0.0012	0.0341	0.0345
448	0.0000	0.1493	0.8507	0.0000	0.0560	0.0560
462	0.0000	0.9838	0.0162	0.0000	0.0053	0.0053
611	0.0034	0.9886	0.0079	0.0034	0.0114	0.0079
618	0.0580	0.9224	0.0196	0.0241	0.0252	0.0068
625	0.3710	0.1643	0.4648	0.1309	0.0412	0.1072
637	0.0544	0.5270	0.4187	0.0200	0.0954	0.0846

Supplementary Table 18
The number of MS/MS spectra that could be detected from each glycosylation site of BG505 mutant (N197D and N276D), related to **Fig. 4c**.

Glycosite	N+0	N+3	N+203	Sum
88	21	1738	783	2542
133	47	540	1364	1951
137	81	1281	646	2008
156	32	260	524	816
160	17	303	344	664
185e	683	972	232	1887
185h	228	1072	178	1478
234	19	257	937	1213
262	0	164	1472	1636
295	0	79	182	261
301	9	81	48	138
332	4	113	298	415
339	54	94	542	690
355	15	1143	396	1554
363	31	201	942	1174
386	0	55	333	388
392	3	41	117	161
398	92	351	200	643
406	0	841	190	1031
411	17	170	869	1056
448	0	22	71	93
462	0	2351	131	2482
611	2	175	14	191
618	176	1166	72	1414
625	877	311	225	1413
637	35	398	140	573

Supplementary Table 19

Site-specific glycosylation of BG505 mutant (N197D and N276D), related to **Fig. 4c**. The proportions of no glycan, complex type, and high mannose glycans that showed significant differences when compared to the glycosylation of BG505 SOSIP.664 trimer purified by Ni^{2+}/SEC shown in **Fig. 1d** were highlighted in purple.

Glycosite	N+0	N+3	N+203	Standard Error (N+0)	Standard Error (N+3)	Standard Error (N+203)
88	0.0028	0.4702	0.5270	0.0010	0.0888	0.0886
133	0.0062	0.1173	0.8765	0.0018	0.0196	0.0205
137	0.0113	0.5676	0.4211	0.0044	0.0527	0.0523
156	0.0031	0.0627	0.9342	0.0015	0.0260	0.0258
160	0.0000	0.1187	0.8813	0.0000	0.0333	0.0333
185e	0.2239	0.6661	0.1100	0.0324	0.0485	0.0211
185h	0.0935	0.8251	0.0814	0.0181	0.0331	0.0227
197	0.0000	0.0000	0.0000	0.0000	0.0000	0.0000
234	0.0009	0.1216	0.8775	0.0003	0.0368	0.0368
262	0.0000	0.0718	0.9282	0.0000	0.0449	0.0449
276	0.0000	0.0000	0.0000	0.0000	0.0000	0.0000
295	0.0000	0.0156	0.9844	0.0000	0.0036	0.0036
301	0.0026	0.0442	0.9532	0.0017	0.0209	0.0202
332	0.0770	0.0871	0.8359	0.0769	0.0756	0.1025
339	0.0128	0.1185	0.8687	0.0082	0.0641	0.0650
355	0.0012	0.6817	0.3171	0.0006	0.0617	0.0616
363	0.0057	0.0991	0.8952	0.0025	0.0601	0.0606
386	0.0000	0.0296	0.9704	0.0000	0.0103	0.0103
392	0.0000	0.0088	0.9912	0.0000	0.0088	0.0088
398	0.1055	0.6074	0.2871	0.0506	0.1002	0.0967
406	0.0000	0.7094	0.2906	0.0000	0.0572	0.0572
411	0.0028	0.1265	0.8706	0.0011	0.0337	0.0339
448	0.0000	0.0000	1.0000	0.0000	0.0000	0.0000
462	0.0000	0.9502	0.0498	0.0000	0.0269	0.0269
611	0.0008	0.9808	0.0184	0.0008	0.0081	0.0075
618	0.1241	0.8494	0.0265	0.0814	0.0805	0.0131
625	0.4757	0.1295	0.3948	0.1504	0.0391	0.1623
637	0.0257	0.4074	0.5669	0.0106	0.0509	0.0458

Supplementary Table 20

The number of MS/MS spectra that could be detected from each glycosylation site of BG505 mutant (N197D, N276D, N301A, and N386D), related to **Fig. 4c**.

Glycosite	N+0	N+3	N+203	Sum
88	48	2933	1300	4281
133	33	1070	1630	2733
137	79	2210	508	2797
156	28	706	769	1503
160	14	887	401	1302
185e	1047	1753	369	3169
185h	291	1948	245	2484
234	12	462	1152	1626
262	3	452	2592	3047
295	24	232	791	1047
332	3	86	388	477
339	62	164	526	752
355	13	1626	362	2001
363	42	1184	1116	2342
392	0	30	133	163
398	64	806	230	1100
406	0	1218	238	1456
411	21	236	1236	1493
448	0	26	66	92
462	0	3250	127	3377
611	1	178	18	197
618	205	950	83	1238
625	766	251	196	1213
637	58	572	160	790

Supplementary Table 21
Site-specific glycosylation of BG505 mutant (N197D, N276D, N301A, and N386D), related to **Fig. 4c**. The proportions of no glycan, complex type, and high mannose glycans that showed significant differences when compared to the glycosylation of BG505 SOSIP.664 trimer purified by Ni^{2+}/SEC shown in **Fig. 1d** were highlighted in purple.

Glycosite	N+0	N+3	N+203	Standard Error (N+0)	Standard Error (N+3)	Standard Error (N+203)
88	0.0125	0.4885	0.4990	0.0076	0.0699	0.0670
133	0.0097	0.0918	0.8985	0.0047	0.0213	0.0231
137	0.0261	0.6102	0.3637	0.0108	0.0740	0.0734
156	0.0029	0.1175	0.8795	0.0018	0.0393	0.0392
160	0.0002	0.1973	0.8026	0.0002	0.0538	0.0538
185e	0.1716	0.7145	0.1139	0.0245	0.0409	0.0280
185h	0.0934	0.8482	0.0584	0.0176	0.0264	0.0150
197	0.0000	0.0000	0.0000	0.0000	0.0000	0.0000
234	0.0007	0.1508	0.8486	0.0004	0.0312	0.0311
262	0.0004	0.1771	0.8225	0.0003	0.0828	0.0830
276	0.0000	0.0000	0.0000	0.0000	0.0000	0.0000
295	0.0012	0.1904	0.8084	0.0005	0.0677	0.0676
301	0.0000	0.0000	0.0000	0.0000	0.0000	0.0000
332	0.0001	0.0401	0.9598	0.0001	0.0310	0.0309
339	0.0094	0.2724	0.7183	0.0045	0.0939	0.0952
355	0.0011	0.6619	0.3370	0.0006	0.0728	0.0728
363	0.0077	0.2253	0.7670	0.0029	0.0952	0.0946
386	0.0000	0.0000	0.0000	0.0000	0.0000	0.0000
392	0.0000	0.1199	0.8801	0.0000	0.1199	0.1199
398	0.0560	0.6369	0.3071	0.0294	0.1137	0.1094
406	0.0000	0.6280	0.3720	0.0000	0.0699	0.0699
411	0.0025	0.2074	0.7902	0.0009	0.0557	0.0560
448	0.0000	0.0247	0.9753	0.0000	0.0126	0.0126
462	0.0000	0.9626	0.0374	0.0000	0.0126	0.0126
611	0.0000	0.9671	0.0329	0.0000	0.0329	0.0329
618	0.2055	0.7721	0.0224	0.1173	0.1170	0.0119
625	0.5364	0.0932	0.3705	0.2438	0.0427	0.2152
637	0.0325	0.4177	0.5498	0.0128	0.0637	0.0555

Supplementary Table 22

The number of MS/MS spectra that could be detected from each glycosylation site of BG505 gp120 monomer, related to **Fig. 4d**.

Glycosite	N+0	N+3	N+203	Sum
88	613	4565	159	5337
133	289	4078	2203	6570
137	1932	4580	370	6882
156	511	1261	1349	3121
160	585	1643	421	2649
185e	2377	2034	70	4481
185h	1103	2563	80	3746
197	418	730	76	1224
234	214	478	1346	2038
262	165	1021	4587	5773
276	53	1374	194	1621
295	43	402	410	855
301	289	170	106	565
332	47	305	592	944
339	411	164	628	1203
355	161	3219	352	3732
363	974	673	1438	3085
386	57	123	194	374
392	0	92	189	281
398	844	776	159	1779
406	187	2425	204	2816
411	1039	513	1355	2907
448	75	63	119	257
462	108	3450	91	3649

Supplementary Table 23

Site-specific glycosylation of BG505 gp120 monomer, related to **Fig. 4d**. The proportions of no glycan, complex type, and high mannose glycans that showed significant differences when compared to the glycosylation of BG505 SOSIP.664 trimer purified by Ni^{2+}/SEC shown in **Fig. 1d** were highlighted in purple.

Glycosite	N+0	N+3	N+203	Standard Error (N+0)	Standard Error (N+3)	Standard Error (N+203)
88	0.2081	0.7603	0.0315	0.0487	0.0546	0.0077
133	0.0707	0.2990	0.6303	0.0207	0.0592	0.0595
137	0.2869	0.5465	0.1666	0.0538	0.0560	0.0386
156	0.0391	0.1600	0.8009	0.0156	0.0264	0.0250
160	0.1272	0.4588	0.4140	0.0567	0.0675	0.0769
185e	0.4194	0.5757	0.0049	0.0473	0.0481	0.0019
185h	0.2352	0.7580	0.0068	0.0358	0.0366	0.0025
197	0.3850	0.5022	0.1128	0.1223	0.1271	0.0755
234	0.0462	0.1717	0.7821	0.0157	0.0409	0.0447
262	0.0078	0.1080	0.8842	0.0032	0.0377	0.0406
276	0.0100	0.7534	0.2366	0.0053	0.0880	0.0855
295	0.0136	0.3413	0.6452	0.0103	0.0655	0.0681
301	0.2849	0.2902	0.4248	0.0800	0.0906	0.0898
332	0.0136	0.0891	0.8974	0.0128	0.0197	0.0225
339	0.1532	0.1825	0.6643	0.0591	0.0697	0.0965
355	0.0273	0.6944	0.2783	0.0093	0.0706	0.0663
363	0.2300	0.0662	0.7037	0.0790	0.0221	0.0848
386	0.0052	0.0894	0.9054	0.0029	0.0365	0.0363
392	0.0000	0.0396	0.9604	0.0000	0.0092	0.0092
398	0.4480	0.4774	0.0746	0.0760	0.0777	0.0180
406	0.0442	0.8316	0.1242	0.0116	0.0241	0.0224
411	0.2157	0.3839	0.4004	0.0434	0.0507	0.0590
448	0.0680	0.1125	0.8194	0.0305	0.0198	0.0216
462	0.0076	0.9761	0.0163	0.0015	0.0052	0.0049

Supplementary Table 24

The number of MS/MS spectra that could be detected from each glycosylation site of JR-FL SOSIP.664, related to **Fig. 5b**.

Glycosite	N+0	N+3	N+203	Sum
88	65	3334	742	4141
135	103	861	771	1735
138	330	1700	282	2312
141	2074	188	89	2351
156	53	410	243	706
160	0	466	98	564
187	14	820	413	1247
241	36	244	99	379
262	32	2531	4526	7089
276	0	321	76	397
295	10	121	374	505
301	25	132	235	392
332	15	169	206	390
339	112	229	92	433
355	0	245	2	247
362	188	251	636	1075
386	0	58	34	92
392	0	78	117	195
397	32	299	21	352
406	33	348	15	396
448	0	25	59	84
463	7	3520	98	3625
611	32	114	0	146
616	163	109	0	272
625	33	865	40	938
637	117	225	95	437

Supplementary Table 25

Site-specific glycosylation of JR-FL SOSIP.664, related to **Fig. 5b**. The proportions of no glycan, complex type, and high mannose glycans that showed significant differences when compared to the glycosylation of BG505 SOSIP.664 trimer purified by Ni^{2+}/SEC shown in **Fig. 1d** were highlighted in purple.

Glycosite	N+0	N+3	N+203	Standard Error (N+0)	Standard Error (N+3)	Standard Error (N+203)
88	0.0030	0.7268	0.2702	0.0011	0.0539	0.0530
135	0.0506	0.3103	0.6391	0.0173	0.0791	0.0755
138	0.3294	0.5651	0.1055	0.0912	0.1014	0.0644
141	0.6274	0.3138	0.0587	0.0982	0.0888	0.0379
156	0.0190	0.1134	0.8676	0.0125	0.0298	0.0256
160	0.0000	0.4754	0.5246	0.0000	0.1362	0.1362
187	0.0008	0.6430	0.3562	0.0004	0.0542	0.0540
241	0.1577	0.2422	0.6001	0.1577	0.0794	0.1389
262	0.0001	0.1743	0.8256	0.0001	0.0853	0.0853
276	0.0000	0.3494	0.6506	0.0000	0.1642	0.1642
295	0.0022	0.1426	0.8552	0.0017	0.0475	0.0475
301	0.0052	0.5568	0.4380	0.0020	0.1113	0.1116
332	0.0088	0.0608	0.9304	0.0069	0.0323	0.0295
339	0.0452	0.2093	0.7454	0.0132	0.0573	0.0490
355	0.0000	1.0000	0.0000	0.0000	0.0000	0.0000
362	0.0281	0.2218	0.7501	0.0128	0.1056	0.1089
386	0.0000	0.5313	0.4687	0.0000	0.1980	0.1980
392	0.0000	0.2953	0.7047	0.0000	0.1231	0.1231
397	0.0012	0.9121	0.0867	0.0007	0.0489	0.0490
406	0.0237	0.9688	0.0075	0.0139	0.0171	0.0036
448	0.0000	0.0606	0.9394	0.0000	0.0338	0.0338
463	0.0003	0.9543	0.0454	0.0002	0.0128	0.0128
611	0.0529	0.9471	0.0000	0.0192	0.0192	0.0000
616	0.8948	0.1052	0.0000	0.0934	0.0934	0.0000
625	0.0000	0.9751	0.0249	0.0000	0.0166	0.0166
637	0.1872	0.1479	0.6649	0.1631	0.0761	0.1557

Supplementary Table 26
The number of MS/MS spectra that could be detected from each glycosylation site of B41 SOSIP.664, related to **Fig. 5c**.

Glycosite	N+0	N+3	N+203	Sum
88	0	2146	508	2654
137	45	101	167	313
140	21	232	43	296
143a	3	282	80	365
156	1	161	447	609
160	0	79	117	196
186e	1	646	156	803
187	195	231	364	790
197	6	254	240	500
234	17	146	554	717
241	12	327	426	765
262	0	175	1817	1992
276	4	69	201	274
295	0	14	156	170
301	3	21	65	89
332	3	22	112	137
339	2	465	87	554
355	4	688	83	775
362	150	117	673	940
386	107	41	668	816
392	0	349	504	853
396	3	208	70	281
413	6	75	546	627
448	0	26	46	72
463	0	949	167	1116
611	546	192	118	856
616	58	954	124	1136
625	456	540	185	1181
637	173	663	406	1242

Supplementary Table 27

Site-specific glycosylation of B41 SOSIP.664, related to **Fig. 5c**. The proportions of no glycan, complex type, and high mannose glycans that showed significant differences when compared to the glycosylation of BG505 SOSIP.664 trimer purified by Ni^{2+}/SEC shown in **Fig. 1d** were highlighted in purple.

Glycosite	N+0	N+3	N+203	Standard Error (N+0)	Standard Error (N+3)	Standard Error (N+203)
88	0.0000	0.5434	0.4566	0.0000	0.0918	0.0918
137	0.1153	0.1342	0.7505	0.0480	0.0399	0.0732
140	0.0453	0.9051	0.0496	0.0208	0.0288	0.0496
143a	0.0000	0.8574	0.1426	0.0000	0.0502	0.0502
156	0.0005	0.0841	0.9154	0.0005	0.0378	0.0378
160	0.0000	0.0667	0.9333	0.0000	0.0435	0.0435
186e	0.0000	0.8791	0.1209	0.0000	0.0306	0.0306
187	0.1116	0.2427	0.6457	0.0290	0.0492	0.0758
197	0.0004	0.6041	0.3955	0.0004	0.1069	0.1070
234	0.0012	0.0549	0.9439	0.0007	0.0193	0.0192
241	0.0003	0.1154	0.8843	0.0002	0.0172	0.0171
262	0.0000	0.0098	0.9902	0.0000	0.0040	0.0040
276	0.0000	0.0230	0.9770	0.0000	0.0091	0.0091
295	0.0000	0.0053	0.9947	0.0000	0.0053	0.0053
301	0.0002	0.0191	0.9807	0.0002	0.0096	0.0097
332	0.0648	0.4662	0.4690	0.0434	0.1361	0.1500
339	0.0002	0.8246	0.1751	0.0002	0.1093	0.1091
355	0.0003	0.8211	0.1786	0.0002	0.0505	0.0506
362	0.0274	0.0258	0.9469	0.0242	0.0148	0.0388
386	0.0142	0.0376	0.9482	0.0032	0.0326	0.0314
392	0.0000	0.2731	0.7269	0.0000	0.0638	0.0638
396	0.0000	0.7532	0.2468	0.0000	0.0890	0.0890
413	0.0021	0.0125	0.9854	0.0015	0.0017	0.0029
448	0.0000	0.0213	0.9787	0.0000	0.0082	0.0082
463	0.0000	0.6138	0.3862	0.0000	0.0836	0.0836
611	0.6547	0.1884	0.1569	0.1285	0.0819	0.0612
616	0.1857	0.6798	0.1345	0.0955	0.1334	0.0583
625	0.7079	0.1674	0.1247	0.1489	0.1065	0.0424
637	0.1008	0.1716	0.7276	0.0434	0.0664	0.0604

Supplementary Table 28
The number of MS/MS spectra that could be detected from each glycosylation site of CRF02_AG_250 SOSIP.664, related to **Fig. 5d**.

Glycosite	N+0	N+3	N+203	Sum
88	8	2508	359	2875
135	23	932	95	1050
141	6	1049	116	1171
156	65	357	283	705
160	15	288	322	625
197	17	428	208	653
241	12	289	82	383
262	3	79	3633	3715
295	0	4	23	27
301	1	20	6	27
332	53	37	45	135
339	52	8	188	248
354	0	216	4	220
386	0	66	76	142
392	2	1	27	30
397	1	107	99	207
401a	0	335	38	373
406	0	361	13	374
411	6	32	300	338
460	24	6625	176	6825
465	1058	3583	2183	6824
611	1	71	6	78
618	27	867	20	914
625	455	170	163	788
637	51	2603	142	2796

Supplementary Table 29

Site-specific glycosylation of CRF02_AG_250 SOSIP.664, related to **Fig. 5d**. The proportions of no glycan, complex type, and high mannose glycans that showed significant differences when compared to the glycosylation of BG505 SOSIP.664 trimer purified by Ni^{2+}/SEC shown in **Fig. 1d** were highlighted in purple.

Glycosite	N+0	N+3	N+203	Standard Error (N+0)	Standard Error (N+3)	Standard Error (N+203)
88	0.0002	0.7983	0.2015	0.0001	0.0346	0.0345
135	0.0041	0.8664	0.1295	0.0018	0.0400	0.0396
141	0.0010	0.8699	0.1291	0.0009	0.0255	0.0255
156	0.0137	0.1100	0.8763	0.0094	0.0208	0.0233
160	0.0012	0.1031	0.8957	0.0005	0.0398	0.0399
197	0.0021	0.7658	0.2321	0.0021	0.1577	0.1556
241	0.0011	0.4318	0.5671	0.0011	0.1466	0.1463
262	0.0000	0.0107	0.9893	0.0000	0.0073	0.0073
295	0.0000	0.0000	1.0000	0.0000	0.0000	0.0000
301	0.0000	0.1229	0.8771	0.0000	0.0531	0.0531
332	0.0470	0.0210	0.9320	0.0299	0.0117	0.0269
339	0.0850	0.0006	0.9144	0.0715	0.0004	0.0713
354	0.0000	0.9969	0.0031	0.0000	0.0031	0.0031
386	0.0000	0.1511	0.8489	0.0000	0.0600	0.0600
392	0.2000	0.0000	0.8000	0.2000	0.0000	0.2000
397	0.0000	0.5265	0.4735	0.0000	0.1919	0.1919
401a	0.0000	0.9700	0.0300	0.0000	0.0300	0.0300
406	0.0000	0.9757	0.0243	0.0000	0.0243	0.0243
411	0.0000	0.0077	0.9923	0.0000	0.0055	0.0055
460	0.0254	0.9311	0.0435	0.0211	0.0224	0.0096
465	0.1586	0.2976	0.5437	0.0446	0.0445	0.0444
611	0.0000	0.9899	0.0101	0.0000	0.0101	0.0101
618	0.0017	0.9961	0.0022	0.0006	0.0011	0.0010
625	0.6335	0.0802	0.2863	0.0757	0.0191	0.0741
637	0.0522	0.6612	0.2866	0.0225	0.0931	0.0988

Supplementary Table 30
The number of MS/MS spectra that could be detected from each glycosylation site of 327c SOSIP.664, related to **Fig. 5e**.

Glycosite	N+0	N+3	N+203	Sum
88	0	852	438	1290
135	6	237	297	540
141	6	384	203	593
156	5	278	232	515
160	120	482	107	709
189	1	473	269	743
197	15	269	452	736
230	0	33	0	33
241	4	133	175	312
262	0	1	185	186
276	2	81	22	105
289	30	64	563	657
301	1	8	25	34
332	19	26	73	118
339	1	7	16	24
355	12	2262	444	2718
386	0	259	62	321
393	0	263	50	313
398	3	59	37	99
413	13	14	64	91
442	0	56	99	155
448	0	13	66	79
461	3	1103	172	1278
465	807	81	390	1278
611	64	79	18	161
616	2	491	111	604
625	579	480	379	1438
637	49	79	40	168

Supplementary Table 31
Site-specific glycosylation of 327c SOSIP.664, related to **Fig. 5e**. The proportions of no glycan, complex type, and high mannose glycans that showed significant differences when compared to the glycosylation of BG505 SOSIP.664 trimer purified by Ni^{2+}/SEC shown in **Fig. 1d** were highlighted in purple.

Glycosite	N+0	N+3	N+203	Standard Error (N+0)	Standard Error (N+3)	Standard Error (N+203)
88	0.0000	0.6728	0.3272	0.0000	0.0512	0.0512
135	0.0009	0.1800	0.8191	0.0004	0.0405	0.0408
141	0.0009	0.4732	0.5259	0.0006	0.0459	0.0460
156	0.0028	0.1364	0.8608	0.0014	0.0644	0.0643
160	0.0000	0.3771	0.6229	0.0000	0.1642	0.1642
189	0.0000	0.5597	0.4403	0.0000	0.1090	0.1090
197	0.0021	0.5440	0.4538	0.0018	0.0999	0.0996
230	0.0000	1.0000	0.0000	0.0000	0.0000	0.0000
241	0.0005	0.0724	0.9272	0.0003	0.0600	0.0599
262	0.0000	0.0000	1.0000	0.0000	0.0000	0.0000
276	0.0101	0.0144	0.9755	0.0000	0.0000	0.0000
289	0.0000	0.0157	0.9843	0.0000	0.0127	0.0127
301	0.0000	0.0087	0.9913	0.0000	0.0000	0.0000
332	0.0123	0.0069	0.9807	0.0071	0.0034	0.0072
339	0.2000	0.4000	0.4000	0.2000	0.2449	0.2449
355	0.0009	0.6451	0.3540	0.0007	0.0781	0.0779
386	0.0000	0.8165	0.1835	0.0000	0.0972	0.0972
393	0.0000	0.7138	0.2862	0.0000	0.1050	0.1050
398	0.0000	0.6815	0.3185	0.0000	0.3185	0.3185
413	0.0000	0.0000	1.0000	0.0000	0.0000	0.0000
442	0.0000	0.0045	0.9955	0.0000	0.0033	0.0033
448	0.0000	0.0264	0.9736	0.0000	0.0000	0.0000
461	0.0213	0.7870	0.1916	0.0213	0.0826	0.0826
465	0.2893	0.0499	0.6608	0.0720	0.0228	0.0697
611	0.4039	0.5186	0.0775	0.0886	0.0725	0.0285
616	0.0000	0.8855	0.1145	0.0000	0.1145	0.1145
625	0.5048	0.4169	0.0783	0.2738	0.2941	0.0783
637	0.0362	0.3602	0.6036	0.0177	0.2164	0.2073

Supplementary Table 32

The number of MS/MS spectra that could be detected from each glycosylation site of SIVcpzMT145 SOSIP.664, related to **Fig. 5f**.

Glycosite	N+0	N+3	N+203	Sum
88	1	600	620	1221
136	143	1369	260	1772
141	314	1473	146	1933
156	14	152	422	588
160	4	130	182	316
186	0	622	136	758
197	5	295	74	374
236	12	42	273	327
241	16	62	197	275
262	9	15	392	416
268	1	129	330	460
301	3	4	3	10
334	0	9	169	178
343	37	58	42	137
356	10	51	101	162
386	1	131	260	392
392	18	35	339	392
397	189	43	65	297
406	38	562	132	732
442	0	35	724	759
448	2	166	558	726
465	22	1823	38	1883
611	288	687	26	1001
616	2	947	99	1048
625	0	221	29	250
637	10	463	10	483

Supplementary Table 33

Site-specific glycosylation of SIVcpzMT145 SOSIP.664, related to **Fig. 5f**. The proportions of no glycan, complex type, and high mannose glycans that showed significant differences when compared to the glycosylation of BG505 SOSIP.664 trimer purified by Ni^{2+}/SEC shown in **Fig. 1d** were highlighted in purple.

Glycosite	N+0	N+3	N+203	Standard Error (N+0)	Standard Error (N+3)	Standard Error (N+203)
88	0.0001	0.4151	0.5848	0.0001	0.0657	0.0657
136	0.0702	0.7246	0.2052	0.0441	0.0868	0.0588
141	0.0407	0.8394	0.1199	0.0162	0.0498	0.0415
156	0.0032	0.1488	0.8481	0.0021	0.0973	0.0970
160	0.0011	0.2175	0.7814	0.0011	0.1577	0.1574
186	0.0000	0.7431	0.2569	0.0000	0.0645	0.0645
197	0.0001	0.7225	0.2774	0.0001	0.1231	0.1231
236	0.0021	0.0190	0.9789	0.0014	0.0091	0.0092
241	0.0014	0.0282	0.9705	0.0014	0.0110	0.0110
262	0.0010	0.0105	0.9885	0.0008	0.0052	0.0050
268	0.0001	0.1534	0.8465	0.0001	0.1069	0.1069
301	0.0920	0.0000	0.9080	0.0000	0.0000	0.0000
334	0.0000	0.0079	0.9921	0.0000	0.0069	0.0069
343	0.0000	0.0276	0.9724	0.0000	0.0089	0.0089
356	0.0098	0.0541	0.9361	0.0098	0.0280	0.0320
386	0.0001	0.1665	0.8334	0.0001	0.0698	0.0698
392	0.0537	0.0100	0.9363	0.0488	0.0050	0.0485
397	0.4521	0.0761	0.4718	0.1213	0.0400	0.1141
406	0.0249	0.6207	0.3544	0.0196	0.0819	0.0829
442	0.0000	0.0156	0.9844	0.0000	0.0086	0.0086
448	0.0001	0.1284	0.8716	0.0001	0.0286	0.0286
465	0.0039	0.9744	0.0218	0.0016	0.0124	0.0113
611	0.1153	0.8641	0.0206	0.0594	0.0631	0.0070
616	0.0037	0.8962	0.1002	0.0027	0.0255	0.0251
625	0.0000	1.0000	0.0000	0.0000	0.0000	0.0000
637	0.0247	0.9716	0.0037	0.0197	0.0199	0.0037
